# Supplementary material for: Selection and transmission of the gut microbiome alone can shift mammalian behavior
Source: Nat Commun. 2025 Oct 27;16:9482. doi: 10.1038/s41467-025-65368-w (PMC12559214; doi:10.1038/s41467-025-65368-w)
Supplement: Supplementary file 1 — Supplementary Information [file 41467_2025_65368_MOESM1_ESM.pdf]

# Supplementary Information

**The PDF file includes:**

Supplementary Materials and Methods (pages 2 - 15)

Supplementary Text (pages 16 – 22)

Figs. S1 to S12 (pages 23 - 35)

References (within Supplementary Information) (pages 36 – 39)

## Material and Methods

### Animals

*Selection and transfer experiments* **-** All animal experiments were performed in accordance with the rules of the State of Baden-Württemberg, Germany, and approved by the Regierungspräsidium Tübingen (Aktenzeichen: EB 02/20M; EB 04/19M). Two wild-derived inbred lines were originally collected from Manaus, Amazonas, Brazil (MAN line) and Saratoga Springs, New York (SAR line)[1]. SAR and MAN inbred lines were transferred from the University of California Berkeley to the Max Planck Institute for Biology, Tübingen, Germany, in the summer of 2018. The lines were maintained in Individually Ventilated Cages (IVCs, Tecniplast) with a nestlet (Zoonlab), plastic hut or tubes (Datesand), and paper-based bedding (ALPHA-dri, SHEPHERD) and were propagated through sib-sib mating for over 20 generations at the time of this study. The animals used in this study were not rederived (*i.e.,* no embryo transfer to an existing laboratory mouse line) and the mouse lines retained population differences in the microbiome in captivity [2,3]. All germ-free C57BL/6NTac mice used in this study were bred and maintained in sterile conditions (isolator bubbles and Tecniplast IsoCages P) at the Max Planck Institute for Biology, Tübingen. To ensure the germ-free status, we tested for contamination monthly by cultivation, microscopy, and/or molecular methods. The facilities were maintained at 22°C with a 12-hour light/dark cycle. Autoclaved water and standard chow (Altromin 1314) were provided *ad libitum*.

*Administrations of Lactobacilli and indolelactic acid* **-** Male C57BL/6J mice (Jax: 000664) used in the gut administration experiments were individually housed under a 12-hour light/dark cycle at the Mount Sinai animal housing facilities and fed a standard (PicoLab 5053) mouse diet. The animals were around 8-12 weeks old and weighed approximately 25-28 grams at

the time of the experiments. They were used in scientific experiments for the first time. This includes no previous exposure to pharmacological agents or alternative diets. The health status of all animals was normal. All animals were individually housed for the experiments. Procedures were approved under Mount Sinai IACUC-2018-0041(de Araujo, PI).

**Fecal transplant experiments from two wild-derived inbred line donors to germ-free recipients**

To characterize the traits of the MAN line and SAR line, we used a total of 14 MAN and 10 SAR mice, all males and singly housed. For recipients, we used male germfree C57BL/6NTac mice, a total of 24 MAN recipients and 26 SAR recipients, with three biological replicates (*i.e.,* each batch included 6-10 recipients per treatment). To simulate the natural transfer of microbes from parents to offspring, we utilized coprophagy. At the time of inoculation, we placed 10–15 fresh fecal pellets from 28-week-old active breeding females of each wild-derived line into the cages of newly weaned germ-free mouse recipients. Male germfree C57BL/6NTac recipient mice were weaned at 3 weeks of age, individuals were randomized from multiple litters, and two individuals were placed per cage with the feces either from the MAN female or the SAR female (MAN-recipients and SAR-recipients, respectively). We conducted this experiment in a span of seven months from the summer to the winter of 2019.

To compare the phenotypes between donors and recipients, we characterized a variety of traits related to morphology, behavior, and metabolism. Recipient's body weights were measured every week (4, 5, 6, 7, and 8 weeks of age, n = 50) and donor body weights were measured bi-weekly (4, 6, and 8 weeks of age, n = 24) (Fig. S1). At 8 weeks of age, the body composition of live animals was measured by EchoMRI (EchoMRI LCC), including fat mass (g), lean mass (g),

and total water mass (g), with a primary accumulation option of three to minimize random errors. To measure behavioral and metabolic traits, animals at 8 weeks of age inside their home cage were moved at 14:00 ± 30 mins into temperature cabinets (26°C) as previously described [44]. Only the lid and food hoppers were replaced by Promethion cage lids (Sable Systems), while the home bedding was left intact to minimize stress on the mice. The recording started at 15:00 ± 30 mins for 24 hours. Cage locations were randomized inside the temperature cabinets for every batch. A single investigator handled all animals and cages. We measured the following traits per 24 hours; food intake (g), water intake (g), energy expenditure (mean kcal/hr using the Weir equation, indirect calorimetry based on oxygen consumption and carbon dioxide production), all activity (All Meters: sum of all movements in meters using the beam break system), and distance traveled (Ped Meters: sum of all direct locomotion in meters with a speed cut off of 1 cm/second using the beam break system). For food intake, water intake, and energy expenditure, we divided the values by body weight to account for body size differences at the time of the measurement. Gas calibration was done before every experiment and mass monitor calibration was done every four months. We used ExpeData software (Sable Systems) to extract the Promethion data using Macro13 (UMC-10.1.13-mouse.mac) and used the 24-hour measurements at five-minute resolution for all downstream analyses.

After collecting the metabolic cage measurements, 8-week-old animals were euthanized by carbon dioxide in their home cage. We collected blood samples by cardiac puncture after euthanasia. The blood was stored at room temperature for 20-30 minutes, centrifuged for 15 mins at 3000 rpm at 4°C, serum was collected, and stored at -80°C until the metabolomics analyses (see below). We also took standard morphological measurements: total length (mm), tail length (mm), hind foot length (mm), and ear length (mm). Body length (mm) was calculated as total

length - tail length. The wet intestinal length was also measured: small intestine (mm), cecum (mm), and large intestine (mm). The cecum contents were stored at -80°C until DNA extraction (see below). Detailed sample information is listed in Supplementary Data 26.

**Selection experiment: Serial fecal transplants from a single donor to selection and control lines.**

*Selection experiment procedure*

Feces from a single adult female SAR donor cage (TAS201) were placed into 16 new cages (10-15 fecal pellets each) as a starter microbial community with sterile bedding, food, and water. Newly weaned male germ-free C57BL/6NTac mice (3-4 weeks of age) were used as recipients (N0 rounds of transfer). Individuals were randomized from multiple litters, placed in cages with the starter microbiome, and separated into two groups (Selection and Control lines, 8 cages each). All animals were singly housed and the cage locations on the IVC rack were randomized. After two weeks, the recipients inside their home cage at 5-6 weeks of age were placed in temperature-controlled cabinets (set to 26°C) at 14:00 ± 30 mins. Phenotyping at 5-6 weeks and measuring at 2 PM allowed a single investigator to perform the work, thereby reducing inter-observer variability, maximizing the number of transfers within the study period, and minimizing disruption during the dark cycle. Only the lid and food hoppers were replaced by Promethion cage lids (Sable Systems) and the recording started at 15:00 ± 30 mins for 24 hours. Cage locations were randomized inside the temperature cabinets. A single investigator handled all animals and cages. After the recording, all animals (N0 rounds of transfer) were euthanized and measurements were taken (see below). For the selection line, feces from two individuals that showed the lowest distance traveled (Ped Meters) were collected. Feces from these individuals

were placed into 4 new cages (10-15 fecal pellets each) with sterile bedding, food, and water (8 cages total for the selection line). For the Control line, the same procedure was performed as the Selection line, but feces from two random individuals (using an online random number generator) were collected instead (8 cages total for the control line). Within an hour of placing the feces into the new cages, newly weaned germ-free recipients (3-4 weeks of age) were placed in the cages, and inoculation occurred via coprophagy (N1 rounds of transfer). We repeated this selection procedure four times (N0 - N4 rounds of transfer). Finally, we repeated the entire procedure in parallel four times resulting in a total of four biological replicates involving a total of 311 germ-free recipient mice from summer to winter 2020.

Promethion data (*i.e.,* food intake, water intake, energy expenditure, all activity, and distance traveled), body weight, body length, tail length, and cecum were collected after two weeks post inoculation (5-6 weeks of age) from all animals involved in the selection experiment. For the first (N0) and last (N4) rounds of transfer, body compositions of live animals were measured at 5-6 weeks of age by EchoMRI (EchoMRI LCC) and serum was collected using cardiac puncture after euthanasia for metabolomics (see below). As described above, gas calibration was done before every experiment, and mass monitor calibration was done every four months. We used ExpeData software (Sable Systems) to extract the Promethion data using Macro13 (UMC-10.1.13-mouse.mac) and used the 24-hour measurements at five-minute resolution for all downstream analyses. Detailed sample information is listed in Supplementary Data 27.

**Confirmation of production of indolelactic acid by *L. johnsonii* LJ10**

*Lactobacillus johnsonii* strain LJ0 was previously isolated on MRS agar plates from the small intestine of a specific pathogen-free housed C57BL/6 mouse [5]. The annotated genome is available in Genbank (assembly ID: GCA_002156645.1). To quantify indolelactic acid (ILA), *L. johnsonii* cultures were divided into cell pellets and filtered supernatant for analysis. *L. johnsonii* was cultured in 10 mL anaerobic MRS medium (n=5) at 37 deg C until stationary phase (15 hr), then centrifuged at 3500 x g for 15 minutes. **Conditioned medium:** The supernatant and MRS medium controls were filtered using a 0.22 µm PVDF membrane to remove residual bacteria, 1 µg/mL ILA D5 was added as internal standard, samples were centrifuged 10,000 x g for 10 min, and the supernatant was collected into the autosampler vial for analysis. **Bacterial pellets:** Each pellet was washed in PBS, centrifuged at 3500 x g for 15 min, and all remaining PBS removed. The bacterial pellets were lyophilized and extracted at a ratio of 11 mg dry pellet to 1 mL solvent (10% water, 90% methanol, spiked with 1 µg/mL ILA D5 as internal standard), with 10 min water bath sonication followed by 2.5 hr vortexing. Cell debris was centrifuged out at 3500 x g for 10 min and the supernatant collected for analysis. ILA was measured using the same method for targeted metabolomics below.

**Administration of *L. johnsonii* to mice and open field tests**

*L. johnsonii* was suspended in sterile water and spread onto MRS agar plates (de Man, Rogosa, Sharpe, Millipore, #110660) containing anaerobic cultivation packs (Thermo Scientific, AnaeroPack™) and incubated at 37°C. The following day, bacterial cells were scraped off from the plates and were harvested by centrifugation at 2500 × g for 5 minutes and diluted in sterile saline to achieve a concentration of approximately $10^9$ cells/mL. Animals were administered

either a saline solution or *Lactobacillus johnsonii* suspension (200 µL) via oral gavage daily for four consecutive days. On the fourth day, after an interval of approximately 4 hours, 10-minutes open field tests were performed separately for each individual mouse. Specifically, animals were placed in a novel Plexiglas arena (Med Associates, 25 cm × 25 cm), where a 150-W lamp positioned above the central subarea was activated to elicit natural aversion to the area, following standard protocol. Animals were tested once in this arena, and average velocity and total distance traveled were calculated using automated video analysis software (EthoVision XT 11.5, Noldus).

We acknowledge that the distance traveled measured by the two systems, the open-field test and Promethion automated cages (Sable Systems), is derived differently. However, the distance and speed data together support the validity of comparing the two. In the one-sided selection experiment using the Promethion system, only movements exceeding a velocity threshold of 1 cm/sec are counted as distance traveled. While EthoVision does not apply a speed threshold by default, it calculates velocity by dividing the distance traveled between frames by the time interval [6], generating a frame-by-frame velocity profile over the test duration. In our study, all animals moved at speeds above the 1 cm/sec threshold (Fig. 5C & 5H), supporting the comparability of the two measurement approaches.

**Intra-duodenum catheterization, indolelactic acid (ILA) infusions, and open field tests**

Mice aged 8–12 weeks (25–28 g) underwent duodenal catheter implantation. Preoperative analgesia (buprenorphine, 0.05 mg/kg, s.c.) was administered 30 minutes prior to induction of anesthesia with 3% isoflurane, followed by maintenance with 1.5% isoflurane. Animals were placed on a thermostatically controlled heating pad (CMA 450; Harvard Apparatus). The abdomen was shaved, disinfected with iodine soap, and sterilized with 70% isopropyl alcohol. A

midline laparotomy was performed to expose the duodenum. A purse-string suture was placed 2 mm distal to the pylorus, into which a 3-mm segment of MicroRenathane tubing (0.025" OD × 0.012" ID; Braintree Scientific) was inserted. The tubing was anchored to the gastric antrum with an additional suture. The tubing was tunneled subcutaneously to the dorsal region and exteriorized through a small incision between the scapulae. The external catheter end was sealed until infusion procedures commenced. The abdominal wall and skin were closed using continuous and interrupted sutures, followed by topical application of Baytril ointment. Postoperative care included infrared heat-assisted recovery and intensive monitoring of locomotor activity and feeding behavior. Analgesia (buprenorphine, 0.05 mg/kg, s.c.) was administered twice daily for three days post-surgery.

**Targeted metabolomics**

We selected twelve metabolites reported to affect host behavior through gut microbiome modulation [4] and measured their concentrations in serum from animals at the start (N0) and end (N4) of the selection experiment using LC-MS analysis performed on a HPLC system (Dionex UltiMate 3000, Thermo Fisher, USA) coupled with a high-resolution mass spectrometer (Impact II, Bruker, Germany). We used the protocol described in [4] for corticosterone, glutamic acid, kynurenic acid and γ-aminobutyric acid (GABA). For the other eight compounds - indolelactic acid (ILA), indoxyl sulfate, indole-3-propionic acid, cortisol, tryptophan, thyroxine (T4), kynurenine and serotonin - an updated LC-MS/MS method was used with the following separation condition: stationary phase C18 Kinetex 2.6 µm; 100 A; 150x2.1 mm kept at 40°C during the analysis. Mobile phase A was water and mobile phase B was acetonitrile, both with the addition of formic acid (0.1%). The following gradient: A/B 99/1 (0 min), 60/40 (10 min),

5/95 (12 to 12.5 min), 99/1 (13 to 15 min) was applied at a constant flow rate of 0.4 ml/min. The injection volume was 5 µl. Kynurenine and serotonin were analyzed in positive ionization while the others were acquired in negative mode. Collision energy was from 15 to 35 eV. Details of sample preparation, controls, settings, and annotation are provided in the published protocol [4].

**Generation and processing of metagenomes**

DNA was extracted from frozen cecal samples using the PowerSoil DNA isolation kit (Qiagen, Valencia, CA, USA) according to the manufacturer's protocol. We prepared metagenomic libraries as described [6] with slight modifications. Briefly, 1 ng of purified gDNA was used in a Nextera (Illumina, San Diego, USA) Tn5 tagmentation reaction to fragment and ligate adaptors in a single reaction, followed by a 14-cycle PCR to add sample-specific barcodes. Libraries were purified, pooled, and quantified. Size selection (400-700 bp) was performed on a BluePippin (Sage Science, Beverly, USA). Libraries were concentrated and further purified as needed using DNA Clean & Concentrator-5 (Zymo Research, Irvine, USA). Sequencing was conducted on a HiSeq 3000 System (Illumina, San Diago, USA) with 150 paired-end sequencing. We used a quality control pipeline described in [6]. Briefly, adapter trimming and quality control filtering were conducted using Skewer 0.2.2 and bbtools "bbduk" command. Reads mapping to the human genome (GRCh37/hg19) and mouse genome (GRCm39) were filtered using bbtools "bbmap" command. The read quality was assessed using Fastqc 0.11.7 and multiQC 1.5a.

**Microbiome analyses**

*Taxonomic and functional profiling*

Functional and taxonomic profiling was conducted using an in house pipeline. Briefly, taxonomic profiling was based on Kraken2 [7] with default parameters, and Bracken v2.2 [8] parameters set to "-t 10 -l S". Functional profiling was based on HUMANn3 v3.0.0.alpha.3 (201901) [9]. Custom databases were created using Struo2 [10] based on GTDB release 207 [11]. The custom database used here is available at (http://ftp.tue.mpg.de/ebio/projects/struo2/GTDB_release207/).

During the initial component-based microbiome data analysis, we rarefied at 150,000 reads, which excluded one sample (R3N5T290) from all downstream analyses. Relative abundances, alpha diversity, and beta diversity were calculated by Qiime2Reproducible, interactive, scalable and extensible microbiome data science using QIIME2 [12]. Observed features, Shannon index, and Faith's PD were calculated for alpha diversity. Bray-Curtis dissimilarity, unweighted- and weighted- UniFrac distances were calculated for beta diversity. Rarefied reads were also used to calculate unstratified and stratified pathways using HUMANn3 v3.0.0.alpha.3 (201901) [9]. To test differences in beta-diversity by groups, we applied PERMANOVA in QIIME2 [12] using the "diversity beta-group-significance" command. To test differences in alpha diversity by groups, we applied the Wilcoxon rank sum test.

For validation purposes, we employed an alternative approach to taxonomic profiling - one based on metagenome-assembled genomes (MAGs). Due to the moderate coverage of the non-rarefied cecal metagenomes, to increase the quality of the assembly they were pooled: for the Experiment 1 - by origin (SAR or MAN) × (donor or recipient) - into 4 metagenomes, for the Experiment 2 - by replicate × round of transfer × treatment (except for all N0 round samples that were pooled into one and the donor's sample that was not pooled) - into 34 metagenomes; yielding 38 pooled metagenomes in total. The metagenomic assemblies, binning into

metagenome-assembled genomes (MAGs), taxonomic classification and dereplication into species-representative genomes (SRGs) were performed as described previously [13], with the MAG quality assessed using CheckM2 [14]. Out of 127 species initially represented by the MAGs, 104 were assigned an SRG as a result of the dereplication; for 11 of the remaining ones that were taxonomically classified at the species level, their representative genomes were downloaded from GTDB. The resulting set of genomes was transformed into a custom reference database for KrakenUniq software [15] that was subsequently used to obtain a taxonomic profile for each non-rarefied metagenome.

*Compositionally aware statistical analysis of microbiome*

For the 310 non-rarefied metagenomes in the selection experiment, the genera with a prevalence <30% at >0.005% relative abundance were discarded, leaving 158 features (the respective number during the species level analysis was 687). The samples outlying (outside of median ± 3 sd) by body weight at inoculation (BWi) or distance traveled were discarded, leaving 303 samples. For the analyses involving metabolomic data, three samples outlying by their metabolomes were also excluded from consideration (leaving 118 samples with both metagenomes and metabolomes). After zero imputation based on Bayesian-multiplicative replacement, per-taxon read counts were CLR-transformed. The distance traveled and scaled serum concentration of each metabolite were adjusted for BWi by collecting the residuals from the respective LOESS model ($\alpha = 0.75$). Compositionality-aware evaluation of the association of each factor of interest to the variation of microbiome composition was carried out by applying PERMANOVA (adonis2 from vegan) to the Aitchison distance matrix using the formula: *beta-diversity ~ distance traveled + BWi + round of transfer + treatment + round of transfer :*

*treatment* + *replicate* (9999 permutations) (Supplementary Data 28). For each significantly associated factor, a compositionality-aware identification of its associated taxa was performed using the Nearest Balance method [16]. The method processes the outputs of a linear model for the normalized taxa abundance values to yield the optimal ratio (balance) of two subsets of taxa (numerator and denominator) associated with the factor. The following formulas were used in the model for obtaining the taxa coefficients for each factor of interest, with clr-transformed abundance values as a response:
~ *adj. distance traveled* + *BWi* + *round of transfer* * *treatment* - for the distance traveled, round of transfer and interaction of treatment with rounds; ~ *adj. metabolite level* + *BWi* + *round of transfer* * *treatment* - for the levels of each metabolite, respectively. The consensus nearest balance was calculated using 100 iterations of cross-validation (train set proportion: 0.67) to include the taxa with reproducibility >80%. Color palettes from the ggsci R package were used for visualization.

**Targeted metabolome analysis**

For each metabolite of interest, its body weight-adjusted scaled value was the input of a linear model (lmer function from lmerTest package) with the following formula: ~ *round of transfer* * *treatment*; the resulting p-values were FDR-adjusted and the findings with p.adj < 0.1 were reported as significant.

## Statistics

*Initial measurements with MAN and SAR donors*

To test differences in trait values between groups (*i.e.,* MAN-donors vs SAR-donors, MAN-recipients vs SAR-recipients), we applied the Wilcoxon rank sum test on raw data. We applied non-parametric tests because the majority of the 16 traits analyzed did not meet the assumptions of normality. Although a few traits were approximately normally distributed, we opted to use a uniform non-parametric approach across all traits for consistency and to take a conservative stance. This also allowed for more comparable interpretation of effect sizes and p-values across traits.

We observed significant effects of body weight at inoculation (3-4 weeks old) and at measurement (8 weeks old) on distance traveled at 8 weeks old, with a stronger correlation for body weight at inoculation (Fig. S2). Given that each batch of newly weaned germ-free mice included multiple litters at slightly different ages, we accounted for both batch and body weight at inoculation. To test whether the differences between groups persist after accounting for body weight at inoculation and batch effects, we created hierarchically nested linear mixed-effects models using "lmer" function in "lme4" R package. Then we conducted model comparisons using likelihood ratio tests using "lrtest" function in "lmtest" R package. We compared two nested models: (model 1) a full model, including a trait of interest as the response variable, groups (*i.e.,* MAN-donors vs SAR-donors or MAN-recipients vs SAR-recipients), and body weight at inoculation (3 weeks of age) as fixed effects, and the batch as random effects and (model 2) a partial model, including all the same variables as the full model except excluding the group variable. The input variables were log-transformed and standardized. Both uncorrected and FDR-corrected p-values are reported (Supplementary Data 1).

*Selection experiment*

To test whether distance traveled significantly differed between treatments (Control vs Selection) or between before and after selection (e.g. N0 vs N4), we applied the Wilcoxon rank sum test on raw data and residual values accounting for covariates (see below). Similar to the pilot experiment above, we compared two nested linear mixed-effects models: (1) a full model, including distance traveled as the response variable, rounds of transfer (N0 vs N1, N0 vs N2, etc.) and body weight at inoculation (3 weeks of age) as fixed effects, and the batch as random effects, (2) a partial model including the same variables as the full model except excluding the round of selection variable. The variables were log-transformed and standardized. AICc (corrected Akaike information criterion) of the models is reported. We compared the models using likelihood ratio tests using "lrtest" function in "lmtest" R package. Both uncorrected and FDR-corrected p-values are reported (Supplementary Data 4).

## Supplemental Text

### Rationale for selection experiment study design

The design of the experiment was inspired by a One-Sided Host-Microbiome Selection experiment [17], where the microbiome is allowed to change in response to selection on a desired trait while the host genome remains constant. For the starting microbial community, we used feces from a single SAR donor. Wild mouse-derived microbiomes are expected to enhance the translatability of laboratory mouse studies to humans [18,19]. Additionally, they are expected to respond more rapidly to selection, as established communities are likely to exhibit minimal initial changes in a new recipient compared to mixed or synthetic microbial starter communities [17].

We chose distance traveled (Ped Meters) as the trait to select for, and in the direction to lower the trait value, for the following reasons. First, activity behaviors showed the strongest evidence of phenocopying among all traits tested, where differences in distance traveled in recipient lab mice reflected that of the donor mice (see Main Text Fig. 1). Second, we selected the high-latitude starter microbiome in the direction from high activity to low activity to reflect the direction of adaptation that occurred in nature, where a higher metabolic rate is likely more beneficial in higher latitudes, and vice versa [20]. Although selecting for low activity has a lower bound (as opposed to selecting for high activity), the pilot experiment indicated that selection could be more effective in reducing the activity than increasing it by considering the natural activity range of the two donor mice (Main Text Fig. 1E).

While selecting the trait in both directions would likely maximize the chance to detect the changes in activity, the lack of a control line that changes stochastically has been criticized in previous studies [17,21,22]. Thus, we decided to characterize the null changes in activity over time

(Control line) to test whether the activity changes observed in the Selection line significantly differed from that of the Control line as in [21,22].

**Wild-derived inbred lines reflect natural variation in host phenotypes and microbiomes**

Wild-derived inbred lines serve as representative models of natural populations in both host phenotypes and gut microbiome composition. First, the wild-derived mice used in the experiment had been maintained in captivity for over 20 generations through sib-sib mating from wild-caught founders and were never rederived (although rederived animals are available through Jackson Laboratory [1]). This inbreeding approach mirrors the standard process used to generate classical inbred mouse lines, and the ability of these animals to reproduce across multiple generations without visible health issues supports the suitability as stable models for studying natural variations in host traits and microbiomes.

Second, key phenotypes (e.g., body weight, body length, and behavior) have been shown to reflect population-level differences across natural populations [3,20], early-generation mice (N1 and N2s) [3,20], and later generations [1], representing natural variation in physiological traits.

Finally, natural variation in the gut microbiome is maintained in captivity for over 10 generations [2], and several microbial lineages show evidence of vertical transmission in these mouse lines [23], further suggesting a stable and representative biological system.

**Microbiome diversity analyses**

To confirm whether we successfully transferred the microbiomes from donors to recipients through coprophagy, we created a beta-diversity PCoA plot based on Bray-Curtis dissimilarity (Main Text Fig. 1F). The PCoA1 axis separates donors and recipients and the PCoA2 axis

separates SAR and MAN microbiomes (PERMANOVA, $F = 15.5$, $p < 0.001$) supporting a successful microbiome transfer. Unweighted- and weighted-UniFrac distances also showed similar patterns (Supplementary Data 3). For the three alpha-diversity metrics tested, none differed between SAR and MAN donors (Supplementary Data 2), and only the Shannon index was significantly reduced in recipients compared to donors (Wilcoxon test $p < 0.025$), indicating a shift in evenness of the community. Together, the results support the idea that population differences in the microbiome can mediate population differences in host traits, especially activity behaviors (Main Text Fig. 1).

**Microbiome metabolic pathway-related analysis**

We also identified microbial pathways from the shotgun metagenomic data that were significantly associated with distance traveled during the selection experiment (Supplementary Data 29&S30). Among 184 unstratified pathways tested, only "gluconeogenesis_III" significantly correlated with distance traveled using the cut-off of $q < 0.25$ accounting for body weight at inoculation and batch effects (Supplementary Data 29). Among the top 10 pathways with the highest coefficients with distance traveled, five pathways are directly involved in cellular respiration ranging from glycolysis, pyruvate oxidation, and TCA cycle (*i.e.*, "gluconeogenesis_III", "pyruvate fermentation to propanoate I", "pyruvate fermentation to acetone", "acetyl-CoA fermentation to butanoate II", and "incomplete reductive TCA cycle"). All five pathways are positively correlated with distance traveled. Among the 829 stratified pathways tested, two pathways remained significant at the cut-off of $q < 0.25$ (Supplementary Data 30). The top pathway was phosphopantothenate biosynthesis I (*Eubacterium* sp.) involved in the biosynthesis of coenzyme A, which is consistent with the unstratified results related to

metabolic reactions involved in cellular respiration. The second pathway was dTDP-L-rhamnose biosynthesis I (1XD8.76 sp.) involved in the biosynthesis of lipopolysaccharides. Stratified pathways linked to *Lactobacillus* or *Limosilactobacillus* were not detected.

**Adjustment of factors for body weight at inoculation for distance traveled, microbiome, and metabolomic analysis**

One known factor that significantly influenced raw distance traveled (Fig. 2B, upper panel) was body weight (Fig. S2). Body weight at both the time of microbiome inoculation (3–4 weeks old) and the time of behavioral measurement (5–6 weeks old) positively correlates with distance traveled (Fig. S2). Because weight gain during this period is influenced by the microbiome, this supports the need to control for body weight only at inoculation (variation due to litter availability) but not at behavioral measurement (variation partly caused by microbiome differences) (Fig. S3).

We also controlled for "batch effects" when interpreting distance traveled data. Each batch represents a combination of replicate number (e.g., R1, R2, R3) and transfer round (e.g., N0, N1, N2) (Supplementary Data 27). A given "batch" refers to the set of multiple litters available at the time when the germ-free mouse cohort (which was always assigned to both control and selection lines) received fecal inoculation at 3–4 weeks of age. Biologically, we interpret "batch effects" as reflecting a combination of maternal environment (e.g., dam age, litter size, reproductive history), time of year (e.g., seasonal variation, changes in animal care staff), time of day (±30 minutes), and cage position (even though positions were randomized).

Examination of the relationship between metabolite levels and body weight at inoculation revealed non-monotonous patterns. Because of their deviations from linear behavior, LOESS (locally estimated/weighted scatterplot smoothing) was preferred to the linear interpolation. For the distance traveled, the deviation was moderate, occurring at the few higher values, therefore, in the non-microbiome/metabolome parts of the analyses, a linear mixed effect model was accepted for its adjustment.

**Assessment of body weight as a mediator of microbiome-induced behavioral change**

We considered the possibility that microbiome-driven behavioral differences may be mediated through changes in body weight. However, three lines of evidence argue against this interpretation. First, body weight at the time of behavioral measurement (5–6 weeks of age) did not change significantly over the course of the experiment for either control or selection lines (N0 vs N4, t-test $q > 0.05$), despite a significant change in body weight at inoculation (3–4 weeks) for the control line (t-test $q < 0.05$) but not the selection line (t-test $q > 0.05$) (Fig. S3). This supports our decision to correct only for body weight at inoculation, which reflects variation due to animal availability in our germ-free facility, rather than weight at the time of measurement, which may itself be influenced by the microbiome.

Second, within each round of transfer, no significant differences in body weight were observed between control and selection lines (Student t-test, $p > 0.05$, Fig. S3), indicating that observed behavioral differences cannot be attributed to treatment-specific differences in body weight. This also highlights a batch effect across rounds, which we accounted for in our statistical model.

Third, in follow-up experiments, inoculation of *Lactobacillus johnsonii* and its metabolite indolelactic acid (ILA) induced behavioral changes in the expected direction without altering body weight or food intake (Fig. 5D, E, I, and J). Taken together, these results support the interpretation that the microbiome influences locomotor behavior independently of body weight.

**Ecological context**

The findings in this study align with previous studies in wild-derived mouse lines, where mice from colder regions display increased wheel running and nest building behaviors compared to those from warmer regions [20,24]. Given the known role of higher physical activity on regulating metabolic responses to cold [25], these findings provide further evidence for microbiome-mediated adaptive host plasticity associated with thermoregulatory adaptation [26–29]. The results suggest that both population differences in the microbiome and host genetics may contribute to maintaining adaptive behavioral variation in wild house mouse populations.

Among the morphological traits, the only significant difference observed in the recipients due to differences in the microbiome was the tail length. Interestingly, a previous study using the same wild-derived inbred lines demonstrated that tail length showed strong plasticity in response to temperature differences, while body weight showed limited plasticity [30]. This suggests that tail length is more responsive to changes in temperature and the microbiome compared to body weight. The contrasting patterns of tail length between the recipients and donors can be attributed to differences in the microbiome's energy extraction capabilities [3,31,32]. The composition and function of the gut microbiome are associated with mouse body mass in the field and captivity: the SAR donors were found to produce higher levels of short-chain fatty acids compared to the MAN donors under the same environmental conditions, with no difference

in food intake [3]. This explains the trend where SAR recipients exhibited greater body weight and longer tail length compared to MAN recipients. The significant opposite patterns of phenocopying for tail length suggest that host genotypic differences primarily determine the allocation of energy to tail growth, with the microbiome playing a secondary role.

**Supplemental figures**

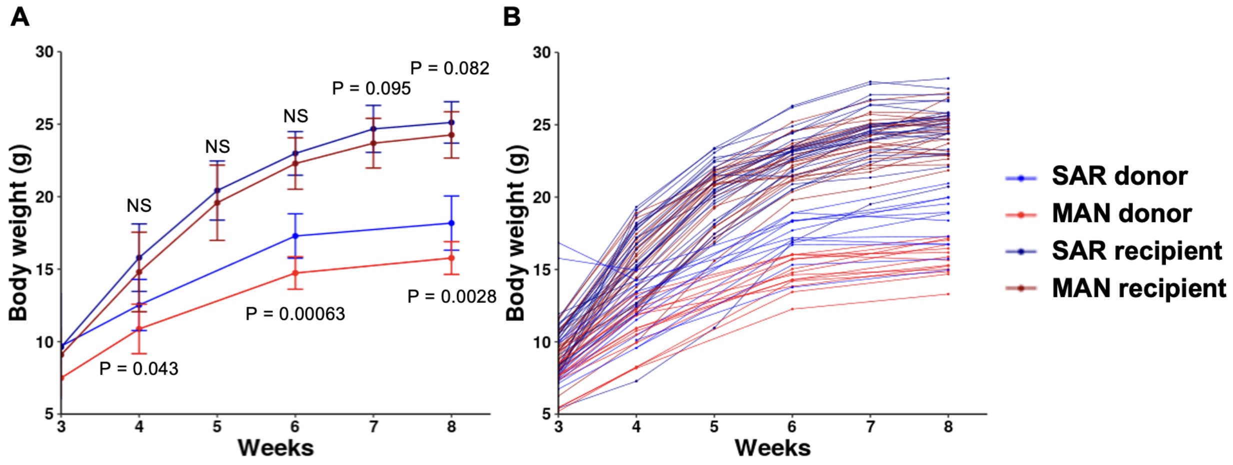


**Figure S1. Growth curves of donors and recipients.** (A) Mean body weights of SAR donors (blue, n = 10), MAN donors (red, n = 14), SAR recipients (dark blue, n = 26), and MAN recipients (dark red, n = 24). Error bars are standard deviations. All animals are male. Wilcoxon rank sum test p-values are plotted for each time point within donors and within recipients: NS p > 0.1. (B) Individual data points for body weight are plotted.

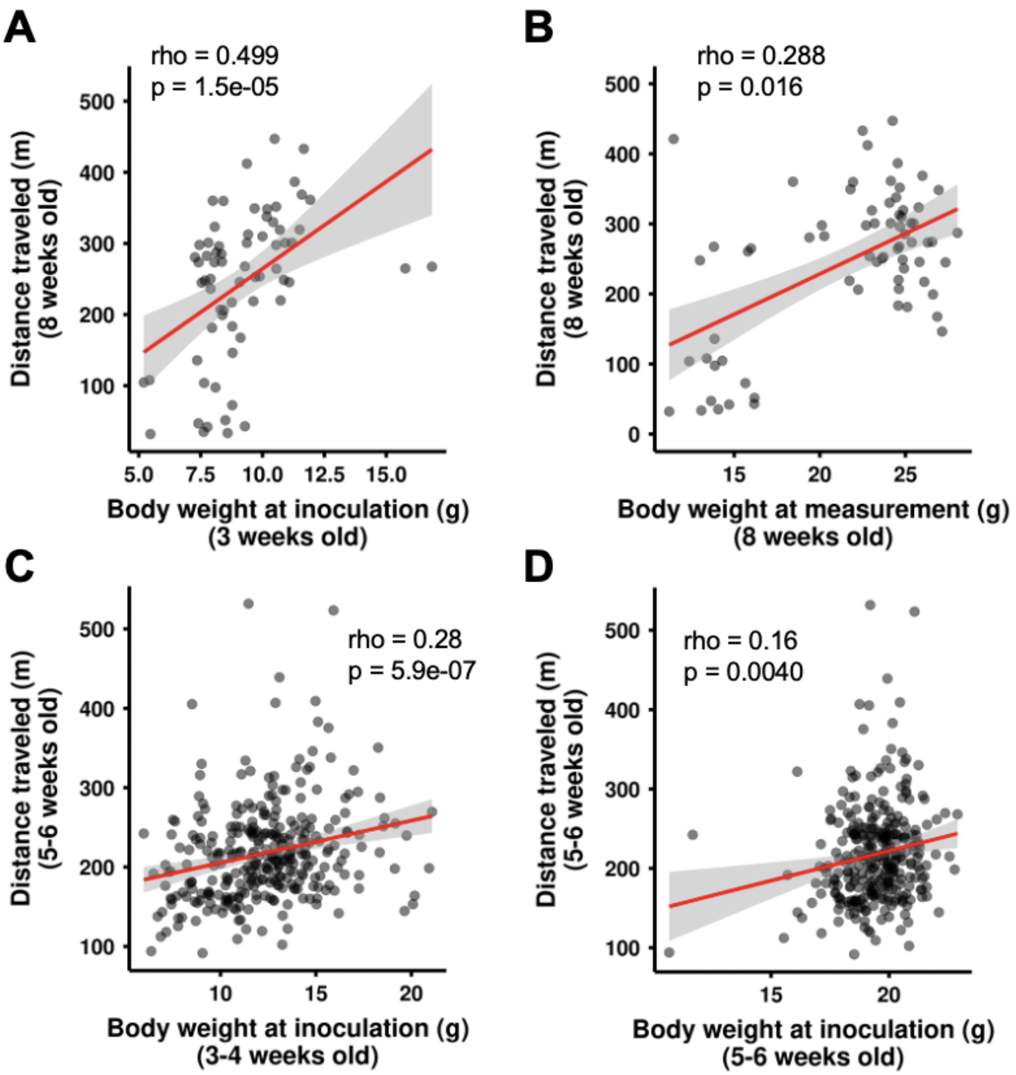


**Figure S2**. Correlations between body weight and distance traveled. Correlations (A) between body weight at inoculation (3 weeks old) and distance traveled (8 weeks old), (B) between body weight at measurement (8 weeks old) and distance traveled (8 weeks old) from the pilot experiment (n = 50). Correlations (C) between body weight at inoculation (3-4 weeks old) and distance traveled (5-6 weeks old), (B) between body weight at measurement (5-6 weeks old) and distance traveled (5-6 weeks old) from the selection experiment (n = 310).

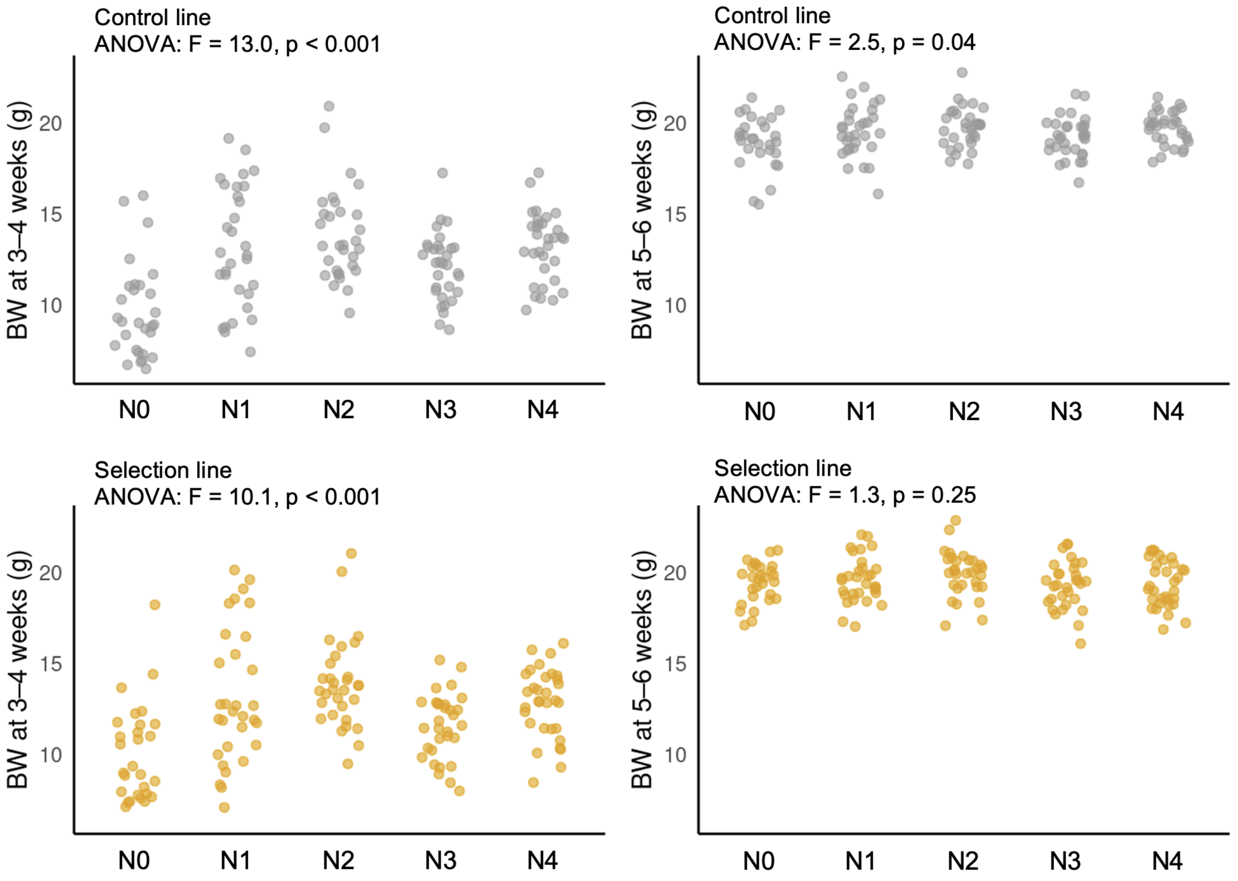


**Figure S3.** Body weight (BW) variation at the time of microbiome inoculation (3–4 weeks old) and distance traveled measurement (5–6 weeks old) in control lines (gray, n = 155) and selection lines (yellow, n = 155). BW variation at 3–4 weeks is primarily due to the availability of newly weaned germ-free C57BL/6 mice. Batch effects are evident across transfer rounds (ANOVA F-values and *p*-values are shown for each comparison), but no significant differences were observed between control and selection lines within any individual round (Student *t*-test $p > 0.05$).

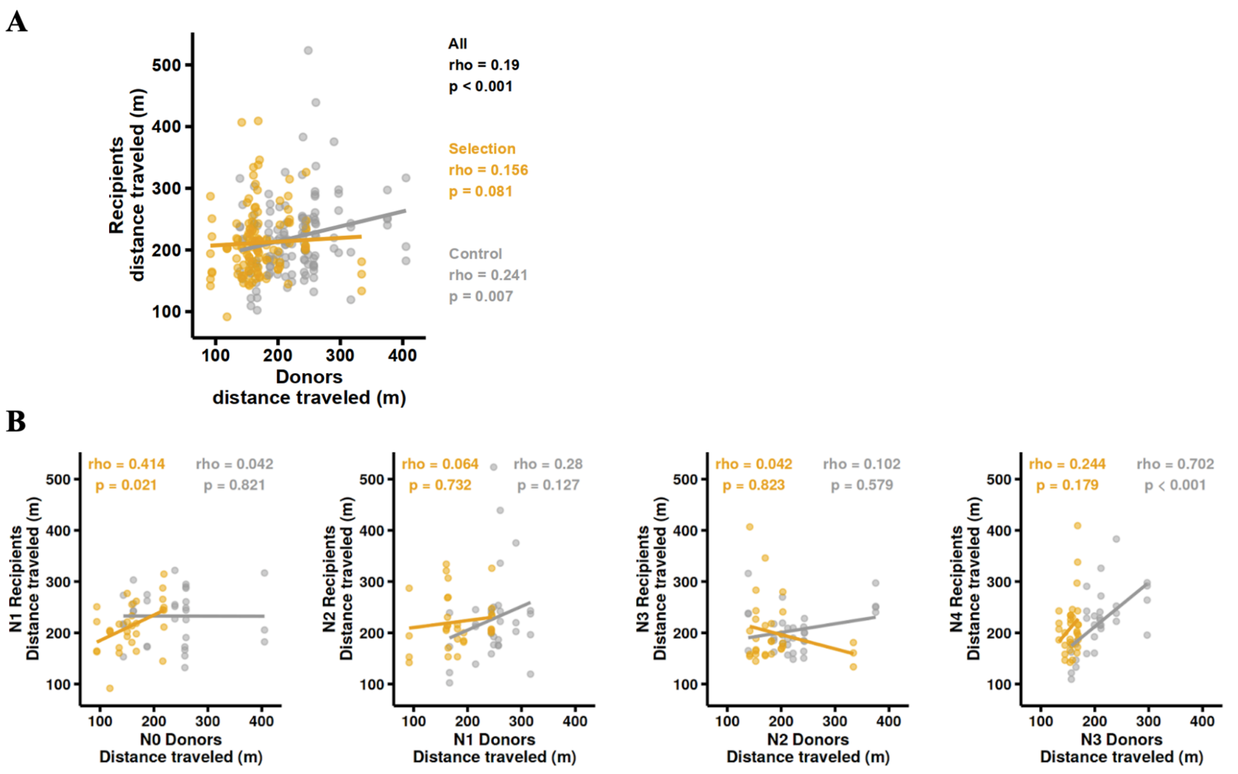


**Figure S4. Community-level heritability of distance traveled.** (A) Correlation of distance traveled between all donors and all recipients. Spearman rho and p-values are shown for all data points (black, N1 – N4, n = 251), Selection line (yellow) and Control line (gray) (B) Correlation of distance traveled between donors (N-1) and recipients (N) by each round of selection (N1: n = 62, N2: n = 62, N3: n = 63, N4: n = 64).

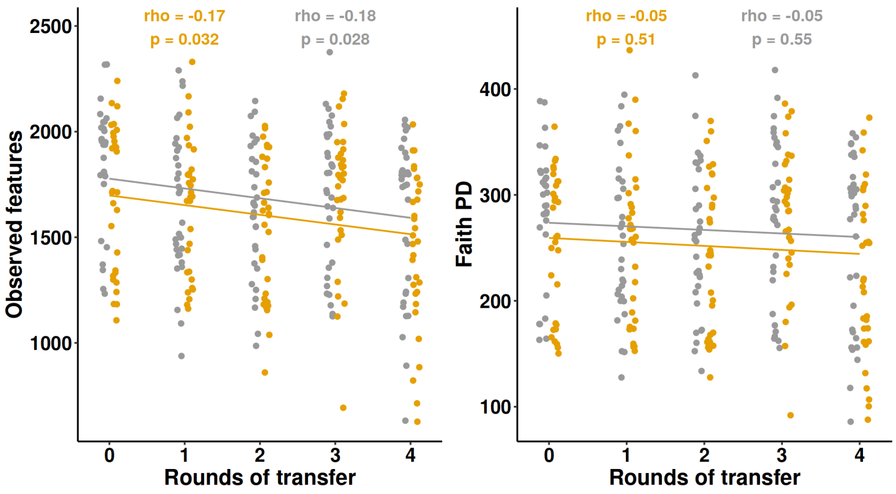


**Fig. S5. Alpha-diversity changes across time.** Observed features showed a significant negative correlation with rounds of transfer. Faith PD did not significantly correlate with rounds of transfer. Colors indicate the selection line (yellow, n = 151) and control line (gray, n = 152). Spearman rho and p-value are shown.

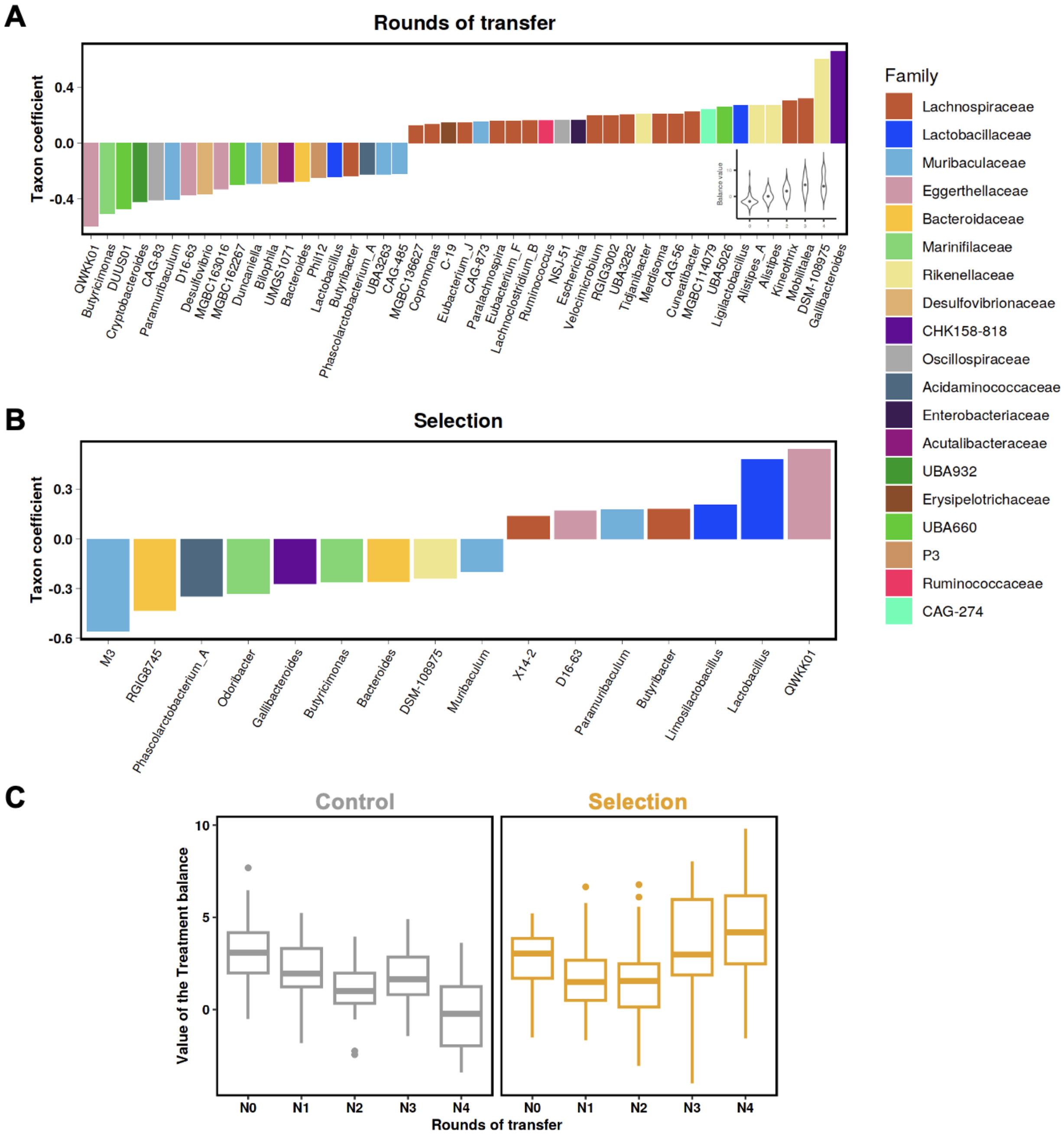


**Figure S6. Nearest balances (NB) associated with the rounds of transfer and selection.** (A) NB associated with rounds of transfer across both Control and Selection group: balance coefficients and its per-sample computed values. Microbial taxa with positive coefficients are enriched in later rounds of selection. (B) NB associated with selection (modeled as interaction of

rounds of transfer and treatment). Microbial taxa with positive coefficients are enriched in the Selection lines, while those with negative coefficients are enriched in the Control lines across the rounds. (C) Dynamics of the selection-associated NB values for Control (gray, n = 152) and Selection lines (yellow, n = 151) across the rounds of transfer.

A

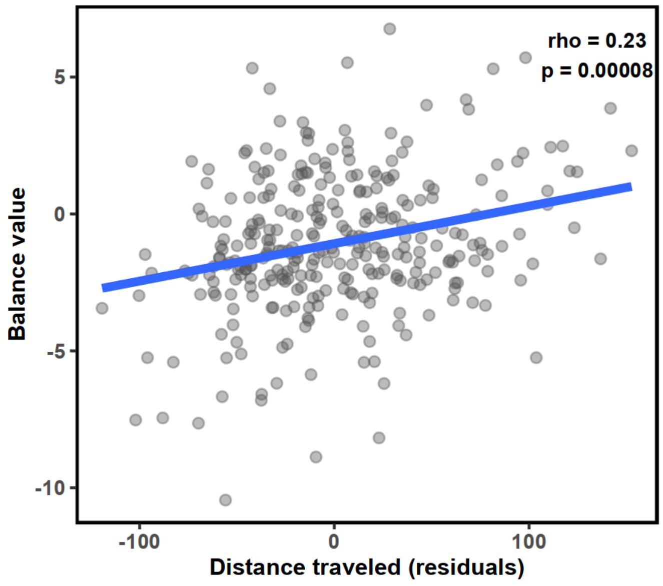


B

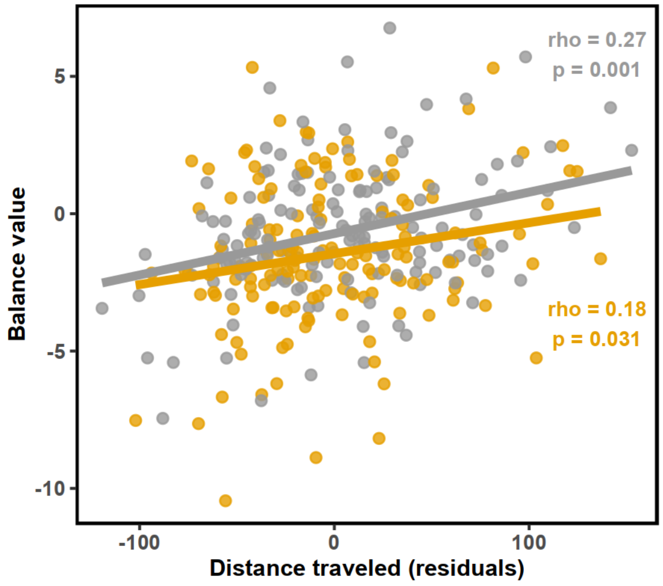


**Figure S7. Correlation of the distance traveled adjusted for the body weight at inoculation with the respective nearest balance values.** Spearman rho and p-value for (A) all data and (B) Control line (gray, n = 152) and Selection line (yellow, n = 151). The figure corresponds to Fig. 3F in the main text.

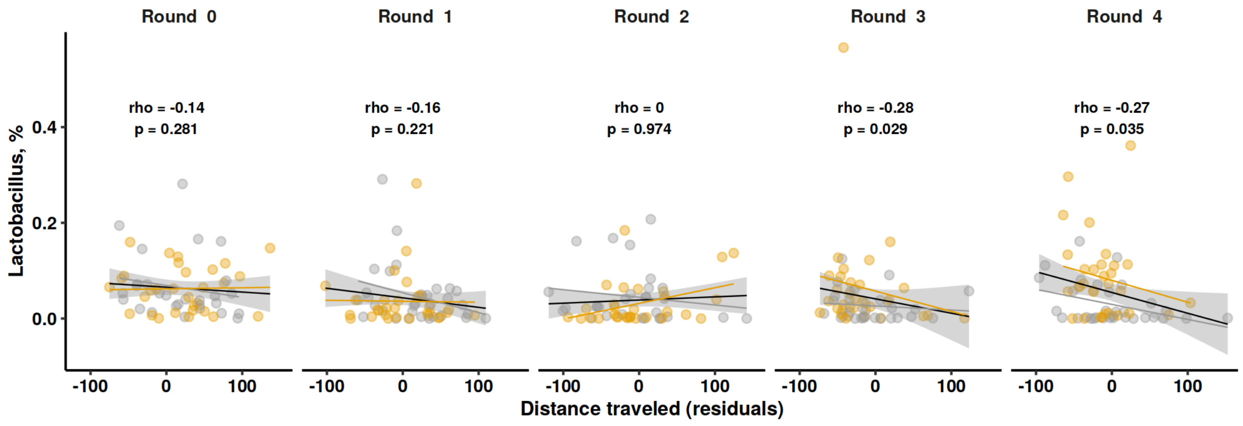


**Figure S8. Correlations between distance traveled adjusted for the body weight at inoculation and relative abundance of *Lactobacillus* across the rounds of transfer.** Colors indicate the Selection (yellow, n = 151) and Control lines (gray, n = 152). The black line shows the linear fit of all data points. Spearman rho and p-value for all data points are shown.

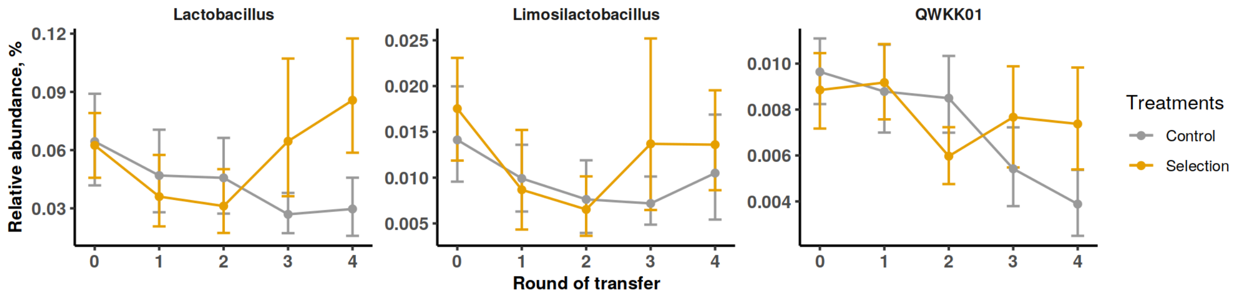


**Figure S9 Abundance dynamics of the genera negatively associated with distance traveled.** The taxa from the denominator of the respective nearest balance are shown; relative abundance values are clr-transformed values. The first panel is from Fig. 3H for comparison purposes. Colors indicate the Selection (yellow, n = 151) and Control lines (gray, n = 152).

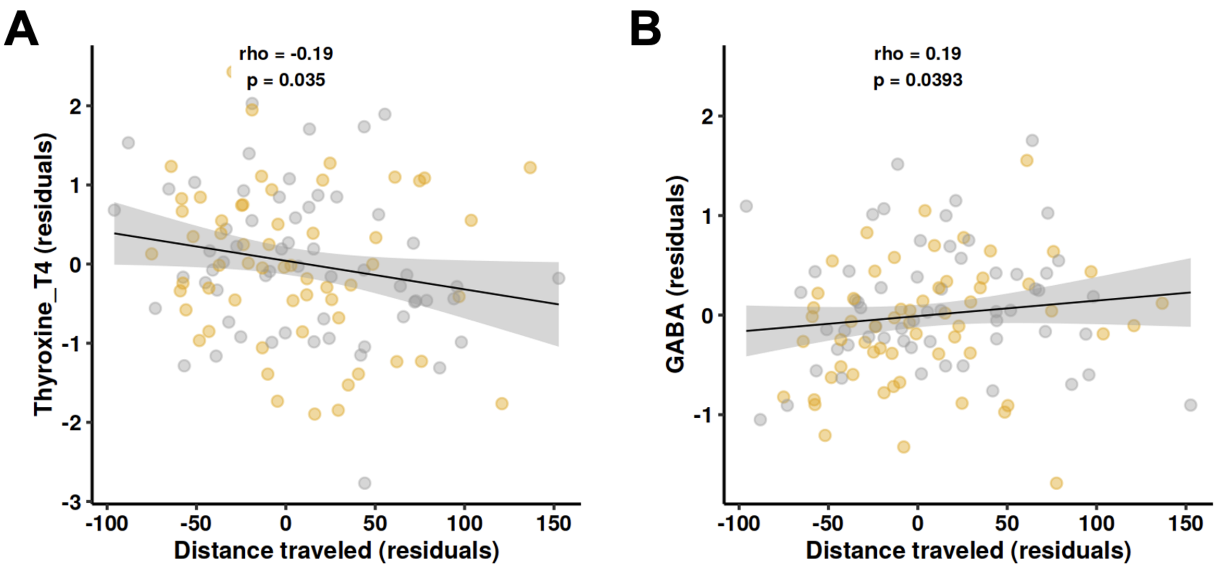


**Figure S10. Metabolites significantly correlated with distance traveled.** (A) Thyroxine and (B) GABA show significant correlations with distance traveled. Spearman's rho and uncorrected p-values are shown (n = 118 samples). A significant negative correlation between distance traveled and Indolelactic acid (ILA) is presented in Fig. 4C. Colors indicate the Selection lines (yellow) and Control lines (gray).

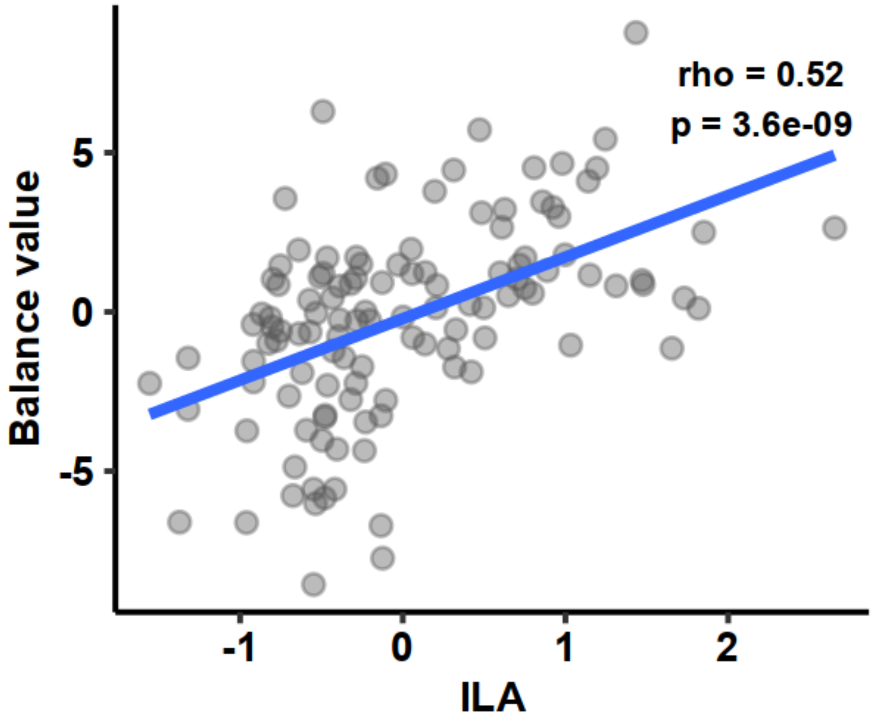


**Figure S11. Correlation of the Indolelactic acid (ILA) adjusted for the body weight at inoculation with the respective nearest balance values.** Spearman rho and p-values are indicated (n = 118 samples). The figure corresponds to Fig. 4D in the main text.

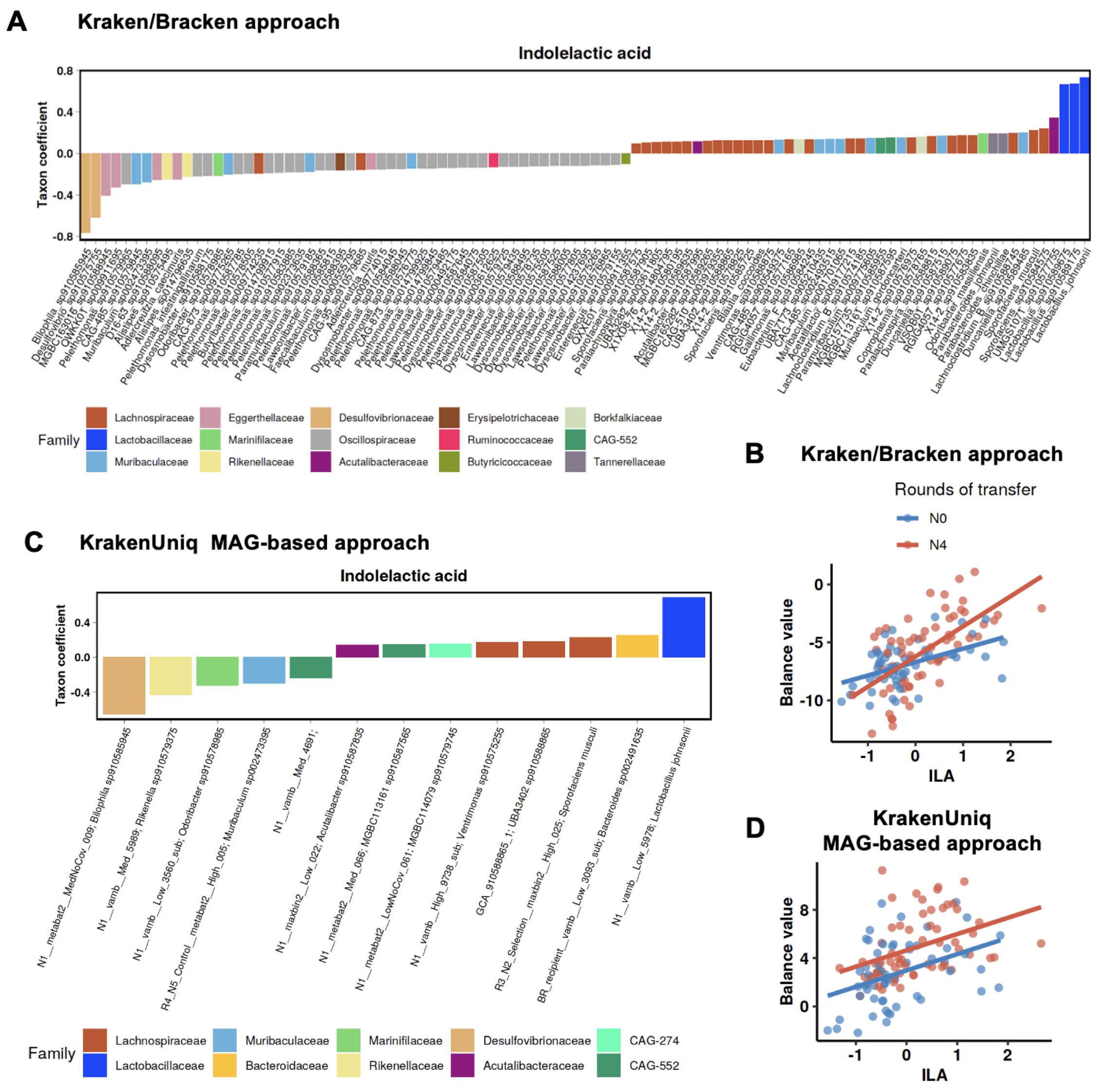


**Figure S12. Species-level nearest balance analysis for indolelactic acid (ILA) reveals similar patterns using two approaches.** The ILA levels had been adjusted for the body weight at inoculation. A) Based on the default taxonomic profiling method (Kraken/Bracken): species-components of the balance, along with its values (B) calculated for each metagenome with a metabolome available (n=118), with the regression lines per round of transfer. C) Based on the MAGs-based taxonomic profiling (KrakenUniq): species-representative genomes (SRG)-components of the balance (including MAGs' internal IDs), along with their values (D).

**References (cited within Supplemental Information)**


1. Dumont, B. L. *et al.* Into the Wild: A novel wild-derived inbred strain resource expands the genomic and phenotypic diversity of laboratory mouse models. *PLoS Genet.* **20**, e1011228 (2024).
2. Moeller, A. H., Suzuki, T. A., Phifer-Rixey, M. & Nachman, M. W. Transmission modes of the mammalian gut microbiota. *Science* **362**, 453–457 (2018).
3. Suzuki, T. A., Martins, F. M., Phifer-Rixey, M. & Nachman, M. W. The gut microbiota and Bergmann's rule in wild house mice. *Mol. Ecol.* **29**, 2300–2311 (2020).
4. Akbuğa-Schön, T. *et al.* The keystone gut species Christensenella minuta boosts gut microbial biomass and voluntary physical activity in mice. *MBio* e0283623 (2023).
5. Di Rienzi, S. C. *et al.* Resilience of small intestinal beneficial bacteria to the toxicity of soybean oil fatty acids. *Elife* **7**, e32581 (2018).
6. Youngblut, N. D. *et al.* Large-scale metagenome assembly reveals novel animal-associated microbial genomes, biosynthetic gene clusters, and other genetic diversity. *mSystems* **5**, e01045–20 (2020).
7. Wood, D. E., Lu, J. & Langmead, B. Improved metagenomic analysis with Kraken 2. *Genome Biol.* **20**, 257 (2019).
8. Lu, J., Breitwieser, F. P., Thielen, P. & Salzberg, S. L. Bracken: estimating species abundance in metagenomics data. *PeerJ Comput. Sci.* **3**, e104 (2017).
9. Beghini, F. *et al.* Integrating taxonomic, functional, and strain-level profiling of diverse microbial communities with bioBakery 3. *Elife* **10**, e65088 (2021).
10. Youngblut, N. D. & Ley, R. E. Struo2: efficient metagenome profiling database construction for ever-expanding microbial genome datasets. *PeerJ* **9**, e12198 (2021).

11. Parks, D. H. *et al.* A standardized bacterial taxonomy based on genome phylogeny substantially revises the tree of life. *Nat. Biotechnol.* **36**, 996–1004 (2018).

12. Bolyen, E. *et al.* Reproducible, interactive, scalable and extensible microbiome data science using QIIME 2. *Nat. Biotechnol.* **37**, 852–857 (2019).

13. Suzuki, T. A. *et al.* Codiversification of gut microbiota with humans. *Science* **377**, 1328–1332 (2022).

14. Chklovski, A., Parks, D. H., Woodcroft, B. J. & Tyson, G. W. CheckM2: a rapid, scalable and accurate tool for assessing microbial genome quality using machine learning. *Nat. Methods* **20**, 1203–1212 (2023).

15. Breitwieser, F. P., Baker, D. N. & Salzberg, S. L. KrakenUniq: confident and fast metagenomics classification using unique k-mer counts. *Genome Biol.* **19**, 198 (2018).

16. Odintsova, V. E., Klimenko, N. S. & Tyakht, A. V. Approximation of a Microbiome Composition Shift by a Change in a Single Balance Between Two Groups of Taxa. *mSystems* **7**, e0015522 (2022).

17. Mueller, U. G. & Sachs, J. L. Engineering Microbiomes to Improve Plant and Animal Health. *Trends Microbiol.* **23**, 606–617 (2015).

18. Rosshart, S. P. *et al.* Wild Mouse Gut Microbiota Promotes Host Fitness and Improves Disease Resistance. *Cell* 1–14 (2017).

19. Rosshart, S. P. *et al.* Laboratory mice born to wild mice have natural microbiota and model human immune responses. *Science* **365**, eaaw4361 (2019).

20. Phifer-Rixey, M. *et al.* The genomic basis of environmental adaptation in house mice. *PLoS Genet.* **14**, e1007672 (2018).

21. Arora, J., Mars Brisbin, M. A. & Mikheyev, A. S. Effects of microbial evolution dominate

those of experimental host-mediated indirect selection. *PeerJ* **8**, e9350 (2020).

22. Mueller, U. G. *et al.* Artificial selection on microbiomes to breed microbiomes that confer salt tolerance to plants. *mSystems* **6**, e0112521 (2021).

23. Sprockett, D. D., Dillard, B. A., Landers, A. A., Sanders, J. G. & Moeller, A. H. Recent genetic drift in the co-diversified gut bacterial symbionts of laboratory mice. *Nat. Commun.* **16**, 2218 (2025).

24. Ferris, K. G. *et al.* The genomics of rapid climatic adaptation and parallel evolution in North American house mice. *PLoS Genet.* **17**, e1009495 (2021).

25. Seebacher, F. & Glanville, E. J. Low levels of physical activity increase metabolic responsiveness to cold in a rat (Rattus fuscipes). *PLoS One* **5**, e13022 (2010).

26. Chevalier, C. *et al.* Gut microbiota orchestrates energy homeostasis during cold. *Cell* **163**, 1360–1374 (2015).

27. Ziętak, M. *et al.* Altered microbiota contributes to reduced diet-induced obesity upon cold exposure. *Cell Metab.* **23**, 1216–1223 (2016).

28. Worthmann, A. *et al.* Cold-induced conversion of cholesterol to bile acids in mice shapes the gut microbiome and promotes adaptive thermogenesis. *Nat. Med.* **23**, 839–849 (2017).

29. Li, B. *et al.* Microbiota depletion impairs thermogenesis of brown adipose tissue and browning of white adipose tissue. *Cell Rep.* **26**, 2720–2737.e5 (2019).

30. Ballinger, M. A. & Nachman, M. W. The contribution of genetic and environmental effects to Bergmann’s rule and Allen's rule in house mice. *Am. Nat.* **199**, 691–704 (2022).

31. Suzuki, T. A. & Worobey, M. Geographical variation of human gut microbial composition. *Biol. Lett.* **10**, 20131037 (2014).

32. Suzuki, T. A. & Ley, R. E. The role of the microbiota in human genetic adaptation. *Science*

**370**, eaaz6827 (2020).
