# Supplementary material for: Selection and transmission of the gut microbiome alone can shift mammalian behavior
Source: Nat Commun. 2025 Oct 27;16:9482. doi: 10.1038/s41467-025-65368-w (PMC12559214; doi:10.1038/s41467-025-65368-w)
Supplement: Supplementary file 2 — Description of Additional Supplementary Files [file 41467_2025_65368_MOESM2_ESM.pdf]

Description of Additional Supplementary Files

File Name: Supplementary Data 1
Description: Mean trait values for wild-derived mouse donors and germ-free laboratory mouse recipients.

File Name: Supplementary Data 2
Description: Median alpha-diversity measurements and pairwise Wilcoxon rank sum test

File Name: Supplementary Data 3
Description: Diversity metrics and statistics

File Name: Supplementary Data 4
Description: Likelihood ratio test and Wilcoxon test results

File Name: Supplementary Data 5
Description: Wilcoxon test results on alpha diversity measurements

File Name: Supplementary Data 6
Description: Pairwise PERMANOVA results on Bray-Curtis dissimilarity

File Name: Supplementary Data 7
Description: Coefficients of the nearest balance associated with rounds of transfer, Genus level, Kraken/Bracken profiling

File Name: Supplementary Data 8
Description: Coefficients of the nearest balance associated with rounds of transfer in the Selection group, Genus level, Kraken/Bracken profiling

File Name: Supplementary Data 9
Description: Coefficients of the nearest balance associated with rounds of transfer in the Control group, Genus level, Kraken/Bracken profiling

File Name: Supplementary Data 10
Description: Coefficients of the nearest balance associated with the selection (interaction of rounds of transfer and treatment), Genus level, Kraken/Bracken profiling

File Name: Supplementary Data 11
Description: Coefficients of the nearest balance associated with the distance traveled, Genus level, Kraken/Bracken profiling

File Name: Supplementary Data 12
Description: Coefficients of the nearest balance associated with the indolelactic acid, Genus level, Kraken/Bracken profiling

File Name: Supplementary Data 13
Description: Coefficients of the nearest balance associated with rounds of transfer, Species level, Kraken/Bracken profiling

File Name: Supplementary Data 14
Description: Coefficients of the nearest balance associated with rounds of transfer in the Selection group, Species level, Kraken/Bracken profiling

File Name: Supplementary Data 15
Description: Coefficients of the nearest balance associated with rounds of transfer in the Control group, Species level, Kraken/Bracken profiling

File Name: Supplementary Data 16
Description: Coefficients of the nearest balance associated with the selection (interaction of rounds of transfer and treatment), Species level, Kraken/Bracken profiling

File Name: Supplementary Data 17
Description: Coefficients of the nearest balance associated with the distance traveled, Species level, Kraken/Bracken profiling

File Name: Supplementary Data 18
Description: Coefficients of the nearest balance associated with the indolelactic acid, Species level, Kraken/Bracken profiling

File Name: Supplementary Data 19
Description: Coefficients of the nearest balance associated with rounds of transfer, Species level, KrakenUniq profiling

File Name: Supplementary Data 20
Description: Coefficients of the nearest balance associated with rounds of transfer in the Selection group, Species level, KrakenUniq profiling

File Name: Supplementary Data 21
Description: Coefficients of the nearest balance associated with rounds of transfer in the Control group, Species level, KrakenUniq profiling

File Name: Supplementary Data 22
Description: Coefficients of the nearest balance associated with the selection (interaction of rounds of transfer and treatment), Species level, KrakenUniq profiling

File Name: Supplementary Data 23
Description: Coefficients of the nearest balance associated with the distance traveled, Species level, KrakenUniq profiling

File Name: Supplementary Data 24
Description: Coefficients of the nearest balance associated with the indolelactic acid, Species level, KrakenUniq profiling

File Name: Supplementary Data 25
Description: Spearman rho correlations between metabolites and distance traveled (over their values adjusted for body weight at weaning)

File Name: Supplementary Data 26
Description: Sample information: Pilot Experiment

File Name: Supplementary Data 27
Description: Sample information: One-Sided Selection Experiment

File Name: Supplementary Data 28
Description: The PERMANOVA test results for the metagenomes of the selection experiment based on the Aitchison distance matrix.

File Name: Supplementary Data 29
Description: Top 20 unstratified pathways associated with PedMeters accounting for body weight at weaning and batch from the selection experiment.

File Name: Supplementary Data 30
Description: Top 20 stratified pathways associated with PedMeters accounting for body weight at weaning and batch from the selection experiment.
