# Supplementary material for: Selection and transmission of the gut microbiome alone can shift mammalian behavior
Source: Nat Commun. 2025 Oct 27;16:9482. doi: 10.1038/s41467-025-65368-w (PMC12559214; doi:10.1038/s41467-025-65368-w)
Supplement: Supplementary file 4 — Reporting Summary [file 41467_2025_65368_MOESM4_ESM.pdf]

nature portfolio

Corresponding author(s): Ruth E. Ley

Last updated by author(s): Jul 7, 2025

Reporting Summary

Nature Portfolio wishes to improve the reproducibility of the work that we publish. This form provides structure for consistency and transparency in reporting. For further information on Nature Portfolio policies, see our Editorial Policies and the Editorial Policy Checklist.

Statistics

For all statistical analyses, confirm that the following items are present in the figure legend, table legend, main text, or Methods section.

n/a	Confirmed	
☐	☒	The exact sample size (*n*) for each experimental group/condition, given as a discrete number and unit of measurement
☐	☒	A statement on whether measurements were taken from distinct samples or whether the same sample was measured repeatedly
☐	☒	The statistical test(s) used AND whether they are one- or two-sided<br>*Only common tests should be described solely by name; describe more complex techniques in the Methods section.*
☐	☒	A description of all covariates tested
☐	☒	A description of any assumptions or corrections, such as tests of normality and adjustment for multiple comparisons
☐	☒	A full description of the statistical parameters including central tendency (e.g. means) or other basic estimates (e.g. regression coefficient) AND variation (e.g. standard deviation) or associated estimates of uncertainty (e.g. confidence intervals)
☐	☒	For null hypothesis testing, the test statistic (e.g. *F*, *t*, *r*) with confidence intervals, effect sizes, degrees of freedom and *P* value noted<br>*Give P values as exact values whenever suitable.*
☒	☐	For Bayesian analysis, information on the choice of priors and Markov chain Monte Carlo settings
☐	☒	For hierarchical and complex designs, identification of the appropriate level for tests and full reporting of outcomes
☐	☒	Estimates of effect sizes (e.g. Cohen's *d*, Pearson's *r*), indicating how they were calculated

Our web collection on statistics for biologists contains articles on many of the points above.

Software and code

Policy information about availability of computer code

Data collection	Promethion behavioral/metabolic data: Sable Systems with Macro13 (UMC-10.1.13-mouse.mac) for beam-break and indirect-calorimetry recordings.<br>Body composition: EchoMRI™ Analyzer software (EchoMRI LCC) for fat/lean mass measurements.<br>Open field: Behavioral data were recorded and analyzed using EthoVision XT 11.5 (Noldus).<br>Metabolomics: Data were acquired using Dionex UltiMate 3000 HPLC (Thermo Fisher) coupled with Impact II mass spectrometer (Bruker).
Data analysis	Metagenomic read quality control and filtering were performed using Skewer 0.2.2, bbtools (bbduk and bbmap), FastQC 0.11.7, and MultiQC 1.5a.<br>Taxonomic profiling was done using Kraken2, followed by Bracken v2.2 with parameters -t 10 -l S.<br>Functional profiling was performed using HUMANn3 v3.0.0.alpha.3 (201901), with custom databases built using Struo2 (based on GTDB release 207).<br>Compositionally-aware analysis was conducted using the NearestBalance R library and vegan (adonis2) in R for PERMANOVA on Aitchison distances.<br>MAGs were assembled and dereplicated using a pipeline described previously, with quality assessment via CheckM2.<br>Alternative taxonomic profiling was conducted using KrakenUniq with custom MAG-based reference databases.<br>QIIME2 was used to calculate alpha and beta diversity metrics and test group differences (diversity beta-group-significance).

For manuscripts utilizing custom algorithms or software that are central to the research but not yet described in published literature, software must be made available to editors and reviewers. We strongly encourage code deposition in a community repository (e.g. GitHub). See the Nature Portfolio guidelines for submitting code & software for further information.

Data

Policy information about availability of data

All manuscripts must include a data availability statement. This statement should provide the following information, where applicable:

- Accession codes, unique identifiers, or web links for publicly available datasets
- A description of any restrictions on data availability
- For clinical datasets or third party data, please ensure that the statement adheres to our policy

Gut metagenomes have been deposited to the European Nucleotide Archive under study accession PRJEB83173. The code used in data analysis is provided provided at GitHub (https://github.com/leylabmpi/sel_trans_behavior/) and Zendo (https://doi.org/10.5281/zenodo.17047502). The metabolomic data were deposited to the MassIVE repository (data identifiers: MSV000097923, MSV000097925, MSV000097928).

Research involving human participants, their data, or biological material

Policy information about studies with human participants or human data. See also policy information about sex, gender (identity/presentation), and sexual orientation and race, ethnicity and racism.

Reporting on sex and gender	*Use the terms sex (biological attribute) and gender (shaped by social and cultural circumstances) carefully in order to avoid confusing both terms. Indicate if findings apply to only one sex or gender; describe whether sex and gender were considered in study design; whether sex and/or gender was determined based on self-reporting or assigned and methods used.* *Provide in the source data disaggregated sex and gender data, where this information has been collected, and if consent has been obtained for sharing of individual-level data; provide overall numbers in this Reporting Summary. Please state if this information has not been collected.* *Report sex- and gender-based analyses where performed, justify reasons for lack of sex- and gender-based analysis.*
Reporting on race, ethnicity, or other socially relevant groupings	*Please specify the socially constructed or socially relevant categorization variable(s) used in your manuscript and explain why they were used. Please note that such variables should not be used as proxies for other socially constructed/relevant variables (for example, race or ethnicity should not be used as a proxy for socioeconomic status).* *Provide clear definitions of the relevant terms used, how they were provided (by the participants/respondents, the researchers, or third parties), and the method(s) used to classify people into the different categories (e.g. self-report, census or administrative data, social media data, etc.)* *Please provide details about how you controlled for confounding variables in your analyses.*
Population characteristics	*Describe the covariate-relevant population characteristics of the human research participants (e.g. age, genotypic information, past and current diagnosis and treatment categories). If you filled out the behavioural & social sciences study design questions and have nothing to add here, write "See above."*
Recruitment	*Describe how participants were recruited. Outline any potential self-selection bias or other biases that may be present and how these are likely to impact results.*
Ethics oversight	*Identify the organization(s) that approved the study protocol.*

Note that full information on the approval of the study protocol must also be provided in the manuscript.

Field-specific reporting

Please select the one below that is the best fit for your research. If you are not sure, read the appropriate sections before making your selection.

☒ Life sciences ☐ Behavioural & social sciences ☐ Ecological, evolutionary & environmental sciences

For a reference copy of the document with all sections, see nature.com/documents/nr-reporting-summary-flat.pdf

Life sciences study design

All studies must disclose on these points even when the disclosure is negative.

Sample size	Sample sizes were based on prior studies using similar experimental designs to assess physiological and microbiome variation. For germ-free mouse studies, sample sizes (typically n=6–12 per group) were selected to balance statistical power and feasibility given the number of treatments and endpoints measured. For the One-sided selection experiment, sample size was determined based on pilot data comparing distance traveled between two wild-derived inbred lines (Fig. 1E). No formal statistical power calculation was performed, but effect sizes in related work guided sample selection.
Data exclusions	No data were excluded from the final analyses unless clearly identified as technical failures (e.g., failed sequencing reactions, low DNA yield or outliers in metabolomic analyses).
Replication	Biological replicates were included for all major experiments. The initial experiment using wild-derived inbred lines included 3 biological replicates per group. The One-sided selection experiment included 4 independent biological replicates per group, enabling robust testing of

evolutionary and physiological hypotheses.

Randomization	Randomization was implemented at multiple levels: DNA extraction well positions, sequencing plate positions, cage positions on the housing rack, and cage order for behavioral Promethion measurements were all randomized to reduce batch effects and environmental confounding.
Blinding	Investigators were not blinded to group assignment for germ-free inoculations or behavioral cage measurements, due to logistical constraints. However, sample identifiers were blinded during DNA extraction and library preparation for sequencing to minimize potential processing bias.

Reporting for specific materials, systems and methods

We require information from authors about some types of materials, experimental systems and methods used in many studies. Here, indicate whether each material, system or method listed is relevant to your study. If you are not sure if a list item applies to your research, read the appropriate section before selecting a response.

Materials & experimental systems

n/a	Involved in the study	
☒	☐	Antibodies
☒	☐	Eukaryotic cell lines
☒	☐	Palaeontology and archaeology
☐	☒	Animals and other organisms
☒	☐	Clinical data
☒	☐	Dual use research of concern
☒	☐	Plants

Methods

n/a	Involved in the study	
☒	☐	ChIP-seq
☒	☐	Flow cytometry
☒	☐	MRI-based neuroimaging

Animals and other research organisms

Policy information about studies involving animals; ARRIVE guidelines recommended for reporting animal research, and Sex and Gender in Research

Laboratory animals	This study involved two wild-derived inbred mouse strains (SAR and MAN), as well as germ-free C57BL/6Tac and C57BL/6J recipient mice. For the one-sided selection experiment, mice were 3–4 weeks old at the start and dissected at 5–6 weeks of age; for the pilot experiment, mice were dissected at 8 weeks. Mice used for Lactobacilli and indolelactic acid administration were 8–10 weeks old.
Wild animals	NA
Reporting on sex	All mice used in this study were male. This choice was made because males experience less stress from single housing compared to females, and to reduce biological variation associated with the estrous cycle. Additionally, using a single sex increased statistical power, as sex-specific effects were not the focus of this study.
Field-collected samples	NA
Ethics oversight	All procedures adhered to German and U.S. regulations (Regierungspräsidium Tübingen EB 02/20M, EB 04/19M; Mount Sinai IACUC-2018-0041), and conducted in accordance with institutional and national guidelines for animal welfare.

Note that full information on the approval of the study protocol must also be provided in the manuscript.

Plants

Seed stocks	*Report on the source of all seed stocks or other plant material used. If applicable, state the seed stock centre and catalogue number. If plant specimens were collected from the field, describe the collection location, date and sampling procedures.*
Novel plant genotypes	*Describe the methods by which all novel plant genotypes were produced. This includes those generated by transgenic approaches, gene editing, chemical/radiation-based mutagenesis and hybridization. For transgenic lines, describe the transformation method, the number of independent lines analyzed and the generation upon which experiments were performed. For gene-edited lines, describe the editor used, the endogenous sequence targeted for editing, the targeting guide RNA sequence (if applicable) and how the editor was applied.*
Authentication	*Describe any authentication procedures for each seed stock used or novel genotype generated. Describe any experiments used to assess the effect of a mutation and, where applicable, how potential secondary effects (e.g. second site T-DNA insertions, mosiacism, off-target gene editing) were examined.*
